# Retrieval of Winter Wheat Leaf Area Index from Chinese GF-1 Satellite Data Using the PROSAIL Model

**DOI:** 10.3390/s18041120

**Published:** 2018-04-06

**Authors:** He Li, Gaohuan Liu, Qingsheng Liu, Zhongxin Chen, Chong Huang

**Affiliations:** 1 State Key Laboratory of Resources and Environmental Information System, Institute of Geographic Sciences and Natural Resources Research, Chinese Academy of Sciences, Beijing 100101, China; lih@lreis.ac.cn (H.L.); liugh@lreis.ac.cn (G.L.); liuqs@lreis.ac.cn (Q.L.); 2 Key Laboratory of Agricultural Remote Sensing, Ministry of Agriculture/Institute of Agricultural Resources and Regional Planning, Chinese Academy of Agricultural Sciences, Beijing 100081, China; chenzhongxin@caas.cn

**Keywords:** leaf area index, PROSAIL, look-up table, GF-1, winter wheat

## Abstract

Leaf area index (LAI) is one of the key biophysical parameters in crop structure. The accurate quantitative estimation of crop LAI is essential to verify crop growth and health. The PROSAIL radiative transfer model (RTM) is one of the most established methods for estimating crop LAI. In this study, a look-up table (LUT) based on the PROSAIL RTM was first used to estimate winter wheat LAI from GF-1 data, which accounted for some available prior knowledge relating to the distribution of winter wheat characteristics. Next, the effects of 15 LAI-LUT strategies with reflectance bands and 10 LAI-LUT strategies with vegetation indexes on the accuracy of the winter wheat LAI retrieval with different phenological stages were evaluated against in situ LAI measurements. The results showed that the LUT strategies of LAI-GNDVI were optimal and had the highest accuracy with a root mean squared error (RMSE) value of 0.34, and a coefficient of determination (R^2^) of 0.61 during the elongation stages, and the LUT strategies of LAI-Green were optimal with a RMSE of 0.74, and R^2^ of 0.20 during the grain-filling stages. The results demonstrated that the PROSAIL RTM had great potential in winter wheat LAI inversion with GF-1 satellite data and the performance could be improved by selecting the appropriate LUT inversion strategies in different growth periods.

## 1. Introduction

Leaf area index (LAI), which is the most important biophysical parameter, represents the total one-half of green leaves area per unit ground surface area (m^2^/m^2^), and therefore is a dimensionless number [1]. It is an indicator of vegetation ecological processes, the canopy interception of light, respiration, photosynthesis, net primary production, transpiration, soil respiration, and energy exchange between the plant canopies and atmosphere [2,3]. Thus, the LAI can be used for precision agriculture, monitoring crop traits and growth status, the reduction of fertilizer usage, assessing crop health, and the improvement of yield prediction [4,5,6]. Due to the significance of describing the properties of the plants and their interaction with the solar radiation and atmosphere [7], the methods of the LAI estimates have provided some valuable means for understanding the physical condition of crops [2,8,9,10]. Currently, the in situ based methods of LAI measurements mainly include either the direct method with destructive sampling, or indirect methods with digital hemispherical photography, the LAI-2000/2200 Plant Canopy Analyzer, or the smartphone app named PocketLAI (Cassandra, Milan, Italy) [11,12,13,14,15]. However, these methods have obvious shortcomings in terms of time consumption, labor input, and economic costing, and it is also difficult to acquire LAI values over large areas [12,16,17,18]. Moreover, it is difficult to capture the high spatial-temporal heterogeneity and variability with the growth of crops via ground measurements [12,19].

Remote sensing provides an effective way to estimate and monitor LAI as it is able to capture the high spatial-temporal heterogeneity and variability of crop LAI, and a large number of studies have been developed that have proven the feasibility of LAI inversion by using remote sensing data [19,20,21,22,23,24]. Generally, there are four main approaches for estimating crop LAI from remotely sensed data [25,26,27,28]: parametric regression, non-parametric regression, physically-based approaches with radiative transfer models (RTMs), and hybrid methods combining physically-based and non-parametric regression methods. The first type of method explicitly determines parameterized expressions relating LAI with a limited number of spectral bands. It is easily carried out and has high computational efficiency by using linear or nonlinear relationships between the in situ LAI and the remotely sensed spectral data. Most studies have commonly used the spectral vegetation indices to minimize the effects of topography, soil background, and atmosphere [29,30,31]. However, these methods tend to be more noise-sensitive when compared to full-spectrum ones, typically depend on the study site, crop type, growth stage, sampling condition and satellite sensor, and are almost impossible to extrapolate to large areas in practical application [32]. In contrast, the second method of non-parametric regression allows for a non-explicit choice on full-spectrum band relationships or fitting functions with LAI. However, most of these methods are considered black boxes, may incur the problem of over-fitting the training dataset, and lead to the statistics deviating from the datasets used for training [28]. The physically-based approaches are based on the RTMs [10] and establish cause-effect relationships between the full-spectrum bands and LAI, which offer an explicit connection between canopy reflectance and plant biochemical and biophysical characteristics, and are suitable for retrieval practical applications with different types of plants [2,33]. The last hybrid method is based on coupling the physically-based RTM methods with advanced non-parametric regression methods. The advantages are that they combine speed, flexibility, and the provision of uncertainty estimates [28]. However, these methods are still in their infancy, and sometimes their practical application may be more difficult. Hence, the physically-based approaches are more generally applicable and promising. Of these methods, the PROSAIL RTM [33], which resulted from the PROSPECT leaf optical properties model [34] coupled with the Scattering by Arbitrarily Inclined Leaves (SAIL) bidirectional reflectance model [35], has become one of the most popular RTMs for the simultaneous estimation of canopy spectral reflectance and LAI [11,26,36,37]. Due to its general stability, robustness, and ease of use, the PROSAIL RTM has also been successfully applied to a variety of agricultural crops at different scales [10,38].

There are various techniques for the inversion of PROSAIL RTM: iterative optimization [33], look-up table (LUT) [10,30], and various machine learning methods (which include artificial neural networks (ANNs) [10,20,39], support vector machines regression [40], genetic algorithms [27], kernel ridge regression (KRR) [41], Gaussian or heteroscedastic Gaussian processes regression [42,43,44,45,46], and some other Bayesian methods [47]). Compared to iterative optimization, the LUT does not take more time to achieve accurate results. When compared to most machine learning methods, the LUT is a general and globally applicable inversion method, which is suitable for any sensor. An additional advantage is that LUT is not considered as a black box and owns a clear inversion process. More importantly for LUT-based inversion methods, the ill-posed inverse problem can be solved by using multiple solutions, available a priori knowledge relating to the distribution of winter wheat variables, adding noise to account for uncertainties attached to models and measurements, land cover classification, and so on [9,10,37,48]. Several studies have found that LUTs have the ability to solve complex inversion problems with PROSAIL models, improved the accuracy and increase robustness of the LAI estimate [2,10,11,49].

Currently, a variety of global LAI products have been produced for vegetation monitoring from various types of satellite sensors, e.g., the Terra/Aqua Moderate Resolution Imaging Spectroradiometer (MODIS) [50,51], and the Système Pour l’Observation de la Terre (SPOT) VEGETATION [31], ENVISAT Medium Resolution Imaging Spectrometer (MERIS) [52], Terra Multi-angle Imaging Spectroradiometer (MISR) [53], and Advanced Very High Resolution Radiometer (AVHRR) [54]. Nevertheless, there is still a large gap in precise agricultural monitoring, which has resulted from the spatial continuity, accuracy, and regional applicability of the coarse spatial resolution of these LAI products. Higher spatial resolutions (10–30 m) and large swath width of remotely sensed images are desperately needed to support the activities of crop growth monitoring and management on a fine scale. In this context, the China High Resolution Earth Observation System (CHEOS) provides valuable higher spatial-temporal information and near-real-time (NRT) observations for crop monitoring. Chinese satellites of High-Resolution Satellite 1 (GF-1) with a swath width of 800 km, coverage repetitive cycle of 4 days, spatial resolution of 16 m, the first satellite of CHEOS, was launched on 26 April 2013. Unfortunately, few studies and testing have been performed for the inversion of crop LAI from GF-1 data.

Therefore, the objectives of this paper were to (1) estimate the regional winter wheat LAI from GF-1 data by inverting the PROSAIL RTM, and account for some available a priori knowledge relating to the distribution of winter wheat characteristics; and (2) explore the effects of different LAI-LUT strategies with reflectance bands and vegetation indexes on the accuracy of winter wheat LAI retrieval with different phenological stages. The remainder of this paper is organized as follows. Section 2 introduces the study areas, field observation data, and remote sensing data. The total inversion schemes, theoretical basis of the PROSAIL model and LUT method, and precision evaluation methods are presented in Section 3.1, Section 3.2, Section 3.3 and Section 3.4, respectively. Section 4 shows the accuracy evaluation of LAI estimates, provides the impact analysis of different LAI-LUT strategies on the accuracy of the LAI retrievals with the different phenological stages, and the ultimate optimal results of LAI inversion. Section 5 discusses the impact factors of LAI retrieval. Finally, Section 6 presents the final conclusions and that the level of winter wheat LAI monitoring operation can be improved by using these obtained results.

## 2. Study Area and Data Preparation

### 2.1. Study Area

**Figure 1:**
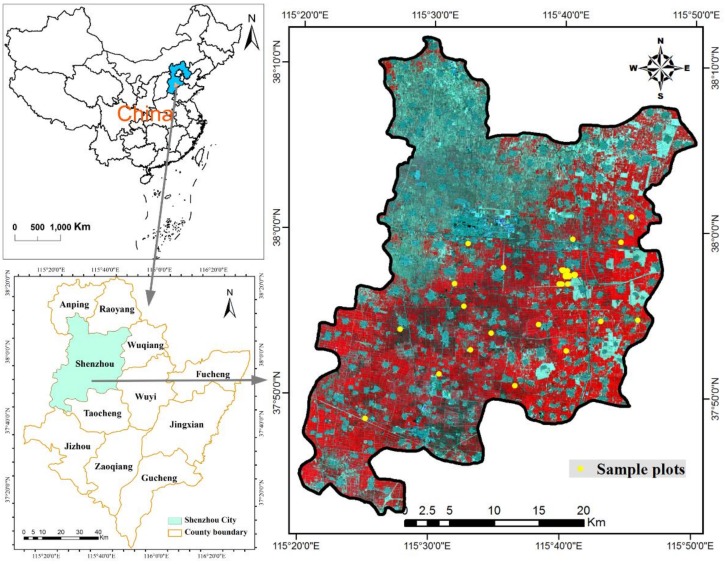
Location of the study area (GF-1 imagery displayed in this figure with false color composite: R = Near Infrared, G = red, B = Green).

Shenzhou City (37°42′–38°11′ N, 115°21′–115°50′ E) belongs to the county-level city in the west of Hengshui City, Hebei Province, China (Figure 1). The study region (which covers approximately 1252 km^2^) is located in the Huang-Huai-Hai Plain of China, which is a major commercial crop planting area dominated by winter wheat and corn in rotation. This area has a warm temperate sub-humid continental monsoon climate with high temperatures and precipitation concentrating in summer, and low temperatures and drought mainly in winter. The soil types mainly consist of the fluvo-aquic soil, wet fluvo-aquic soil, and sandy fluvo-aquic soil. Due to the suitable conditions of soil, climate, and adequate irrigation from groundwater, this study area is suitable for growing winter wheat, cotton, and corn. The terrain of this area is alluvial plain, and the topography owns the characteristics of being relatively flat and tilts slightly from the southwest to northeast. The average elevation ranges from 16 m to 29 m. The area of winter wheat occupies 667.53 km^2^ (approximately 53.32% of the total area). According to our field observations in this region, winter wheat is usually sown in early October and harvested in early or mid-June of the following year.

### 2.2. Field Observation Data

Field samplings were carried out from 17–19 March (green-up), 9–12 April (elongation), 5–7 May (heading), and 27–30 May (grain-filling) in 2015. First, 44 sample plots of homogenous wheat areas were selected from the spatial distribution of winter wheat in 2015, which was derived from the Key Laboratory of Agricultural Remote Sensing, Ministry of Agriculture. To avoid the influence of construction land, trees, or other land types, the samples were at least 100 m away, and each sample was approximately 16 m × 16 m, with three 5 m × 5 m areas measured in each plot. The exact positions of the sample plots were determined using a Trimble GeoXT3000 GPS (Trimble, Sunnyvale, CA, USA).

The in situ-based LAI measurements mainly include direct and indirect methods [11,12,13,14,15]. Compared to the direct methods, the indirect methods do not require destructive harvest, measure faster, and allow a larger spatial sample collection [14]. Classical commercial instruments for indirect LAI estimation with digital hemispherical photography or LAI-2000/2200 Plant Canopy Analyzers (LI-COR, Inc., Lincoln, NE, USA) have been widely used. Recently, a smartphone app called PocketLAI was also presented for LAI measurements [14]. Among these indirect techniques, LAI-2000/2200 Plant Canopy Analyzers have proven to be a good alternative to destructive methods in many experimental conditions. Thus, a LAI-2200 plant canopy analyzer was used to measure the wheat LAI non-destructively under diffuse illumination conditions [55]. The wheat LAI was observed based on one above-canopy and four below-canopy measurements of the incoming radiation in each field. To avoid the influence of direct sunlight on the sensor and minimize the effects of the illumination and boundary conditions, a 45° view cap was used to limit the azimuthal field of view, facing away from the operator [49]. Due to the small influence of non-photosynthetic plant components (almost non-existent) or leaf clumping, the measured LAI was not corrected in this study. Finally, the unique LAI was received from averaging the three areas in each sample.

The leaf chlorophyll content (Cab) was measured using two methods. First, the non-destructive method was measured using a soil and plant analyzer development (SPAD) 502 chlorophyll-meter. Three layers with the base, middle, and top part of the wheat were selected at each point where ten leaves were measured, and three measurements of the SPAD readings were averaged. Finally, the unique Cab was obtained by averaging the three areas in each sample. Second, to transform the SPAD readings (unitless) to absolute leaf chlorophyll contents (μg·cm^−2^), the extraction of leaf chlorophyll content from the 18 representative samples was simultaneously performed in the laboratory during each growth period. After the SPAD measurements, the wheat was then destructively sampled, and all green leaves were collected and brought into the laboratory. For fresh green leaves, each sample was divided into three parts, placed in 95% ethanol separately, and stood for 24 h in the dark. Then, based on the standard methods, the leaf chlorophyll contents were measured using a colorimetric spectrophotometer [9]. Finally, the absolute leaf chlorophyll contents were received from averaging each of the three parts. Furthermore, the specific transfer functions of winter wheat were obtained from these 72 samples as follows:

Cab = 5.6351 **×** exp (SPAD **×** 0.0287 + 0.8095) (R^2^ = 0.9718) (1)

### 2.3. Remote Sensing Data

In this study, the optical remotely sensed images of the GF-1 Wild Field Camera (WFV) were derived from the China Centre for Resources Satellite Data and Application (CRESDA), and Table 1 shows the characteristics of the WFV sensor. The spatial resolution, revisit period, and swath width of this sensor were 16 m, 4 days, and 800 m, respectively. When compared with the commonly available data of MODIS and Landsat, the GF-1 data own good spatial-temporal resolution and are suitable to obtain more optical images to invert the wheat LAI at a finer scale in Shenzhou City. In this study, two high quality images covering the study region on 14 April and 25 May in 2015 were acquired after considering the presence of cloud/smog and facilitating the accuracy validation of the inversion results, and the detailed image information was listed in Table 2.

**Figure 2:**
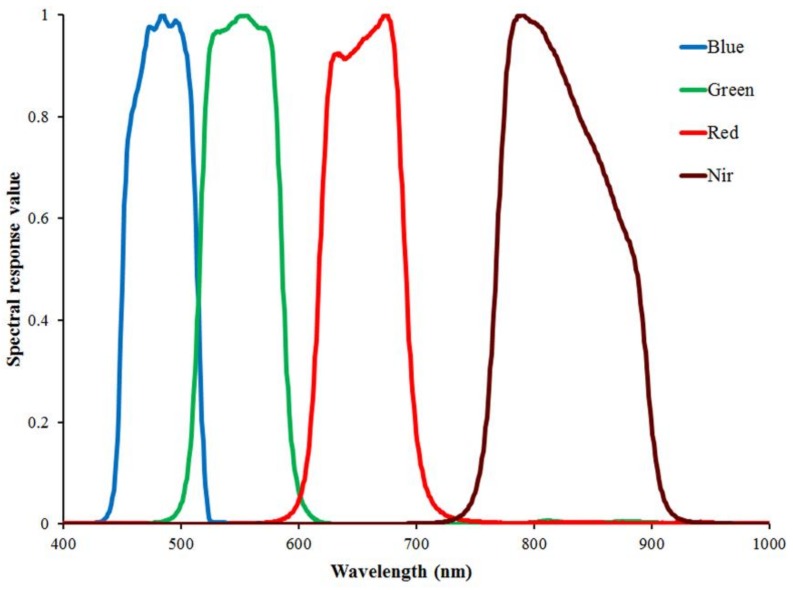
Spectral responses functions of the blue, green, red and near-infrared bands for GF-1 WFV1.

A series of pre-processing for GF-1 images was performed using the ENVI 5.1 software that mainly included radiometric calibration, atmospheric correction, and geometric correction. The apparent radiance was calculated from the digital number (DN) with the calibration coefficients from the CRESDA (Table 1). The atmospheric correction was performed by the fast line-of-sight atmosphere analysis of spectral hypercubes (FLAASH) model in the ENVI software, which includes a RTM of moderate resolution atmospheric RTM (MODTRAN) 5.0 [56]. The required spectral response functions were also from the CRESDA website (Figure 2). The geometric correction was performed with three steps. First, ortho-rectification was performed using the geo-location attached to the GF-1 images and digital elevation model (DEM) with a 30 m resolution. Then, using 30 ground control points (GCPs) collected from Trimble GeoXT3000 GPS observations, the preliminary geometric images were geometrically rectified. Finally, the corrected image from 14 April was used to rectify the other image from 25 May, and the final registration accuracy was less than half a pixel. In the geometric correction, the second-order polynomial transformation with the nearest-neighbor method was used to resample the images in the same resolution. The projection and coordinate system were set as the Universal Transverse Mercator Grid System (UTM) and the World Geodetic System 1984 (WGS84), respectively [11].

## 3. Method

### 3.1. Inversion Schemes

**Figure 3:**
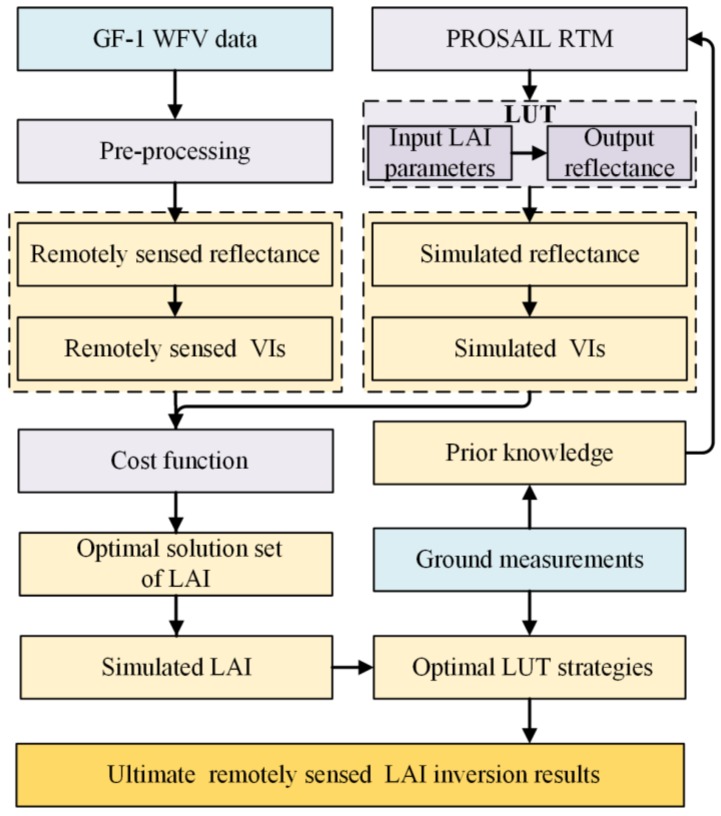
Flow chart of remotely sensed winter wheat leaf area index inversion.

The inversion flow chart is shown in Figure 3. Four steps were undertaken in this process. First, the fit between simulated and measured wheat LAI was assessed based on the reflectance and vegetation indices (VIs). To avoid spectral band information repetition, 15 combinations of four spectral bands were selected. Although the blue band showed vulnerable effects to the residual atmosphere, it is also the chlorophyll sensitive band, which eventually influences the LAI estimates. Therefore, this band was also considered to make full use of the spectral information in this study. To make full use of the typical characteristics of the vegetation spectra, 10 types of VIs were selected according to the strong correlation between the LAI and VIs (Table 3 and Table 4). Second, based on the generated LUT in conjunction with the remotely sensed spectral bands, VIs, and the PROSAIL model, the inversion process was realized. The third step involved the inversion of the LUT to achieve the simulated LAI. Finally, validations were used to achieve the optimal LUT strategies and obtain the ultimate LAI estimates based on the ground measurements. Prior knowledge derived from the ground measurements was also used to remove the influence of ill-posed inversions in this procedure.

### 3.2. PROSAIL Model

PROSAIL RTM, a combination of the PROSPECT [33] and SAIL models [35], was used to retrieve the winter wheat LAI in this study. The PROSPECT model is used to simulate the leaf directional-hemispherical reflectance and transmittance as the function of four biochemical and structural parameters at the leaf level: leaf structure parameter (*N_i_*), leaf chlorophyll *a + b* concentration (Cab), equivalent water thickness (*C_w_*), and dry matter content (*C_m_*). The SAIL model is used to simulate the top of the canopy reflectance as a function of a series of parameters: canopy parameters of *LAI*, average leaf inclination angle (*ALIA*) and hot-spot parameter (*hot*); soil parameter of soil brightness parameter (*psoil*); solar parameters of fraction of diffuse incoming solar radiation (*skyl*) and sun zenith angle (*tts*); and the sensor parameters of sensor viewing angle (*tto*) and relative azimuth angle (*psi*) between the sensor and sun.

As there are differences in the distribution of leaf and canopy parameters among the different crop types with small differences of the soil and solar parameters, the parameterizations of the ranges and distributions of some leaf and canopy parameters (e.g., LAI, Cab, *C_w_*, *C_m_*, and *ALIA*) were set as the prior knowledge from ground measurements. Other parameters were similar to previous studies with wheat, and all ranges and distributions of the variables were selected and set based on these studies [2,10,25,27,48,68,69,70,71]. Due to the small influence on wheat reflectance, the parameter *skyl* was fixed at 0.1, in accordance with previous studies [10,11,32]. Corresponding to the satellite transit time, the solar and sensor parameters could be read directly from the header files of the GF-1 images. All of the input variables are summarized in Table 5.

### 3.3. Look-Up Table (LUT)

The LUT is a conceptually simple method that relies on a large multiple-solutions database for the retrieval of LAI. It has the potential to handle the complex RTM of PROSAIL and increase the robustness of the LAI estimates. Compared to the iterative optimization algorithm, it does not need to consume a significant amount of time. It is also a general and globally applicable method that owns a clear inversion process when comparing it to the method of the neural network with the characteristic of a black-box [37].

To build the LUT, the input parameters were randomly generated to simulate the broadband reflectance of GF-1 WFV. The ranges and distributions for each parameter and the spectral response functions should also be considered. To achieve an appropriate representation, a large database with a total number of 100,000 reflectance/VIs variables [11,32,49] was generated. However, this large number of LUT did not solve the problem that some different variable combinations may own a similar response of radiometric signals or where similar variables may own different responses of radiometric signals in the LUT [72]. To overcome these ill-posed inversion problems, the LUT solution can be calculated by averaging similar parameter combinations with the smallest differences between the remotely sensed reflectance/VIs and the simulated reflectance/VIs [11,32,72]. The relative root mean square error (*RRMSE*) was used as the cost function in this study, and is defined as follows:

(2) $$RRMSE_{R}=\sqrt{\frac{1}{m}\overset{m}{\underset{i=1}{\sum }}{\left(\frac{R_{RS,i}-R_{Simulated,i}}{R_{RS,i}}\right)}^{2}}$$

(3) $$RRMSE_{VI}=\sqrt{{\left(\frac{VI_{RS,i}-VI_{Simulated,i}}{VI_{RS,i}}\right)}^{2}}$$

where *R_RS_* is the remotely sensed band reflectance; *R_Simulated_* is the simulated reflectance; *VI_RS_* is the remotely sensed VI; *VI_Simulated_* is the simulated VI, which was calculated from the simulated reflectance; and *m* is the number of spectral bands.

The solution is a set of variables corresponding to the reflectance/VIs in the LUT that minimizes the RMSE value. However, due to measurement errors and model inadequacies, the solution may not be the unique and optimal result. To overcome these ill-posed inversion problems, a solution with a threshold of 10% of the sorted smallest *RRMSE* was selected and averaged these parameter combinations in the LUT size of 100,000. The 10% threshold was feasible for the accuracy of the LAI estimates, balancing the computer resources, and was consistent with the optimal solutions’ number adopted by previous studies [10,11,32,68].

### 3.4. Precision Evaluation

To fairly compare the different variable combinations of the LAI estimates, 44 sample plots were used for the accuracy assessments. The *RMSE* (kg/ha) and coefficient of determination (R^2^, unitless) were used to validate the estimated LAI results, and evaluate the performance of the LUT strategies using PROSAIL RTM with GF-1 WFV data. The formulas are defined as follows:

(4) $$R^{2}={\left(\frac{n\sum_{i=1}^{n}y_{i}y_{obs,i}-\sum_{i=1}^{n}y_{i}\sum_{i=1}^{n}y_{obs,i}}{\sqrt{n\sum_{i=1}^{n}{\left(y_{i}\right)}^{2}-{\left(\sum_{i=1}^{n}y_{i}\right)}^{2}}\sqrt{n\sum_{i=1}^{n}{\left(y_{obs,i}\right)}^{2}-{\left(\sum_{i=1}^{n}y_{obs,i}\right)}^{2}}}\right)}^{2}$$

(5) $$RMSE=\sqrt{\frac{\sum_{i=1}^{n}{\left(y_{i}-y_{obs,i}\right)}^{2}}{n}}$$

where *y_i_*, *y_obs_*_,*I*_, and *n* are the simulated values, observational values, and number of sample plots, respectively.

## 4. Results

### 4.1. Effects of Different LAI-B Strategies on LAI Retrieval

**Figure 4:**
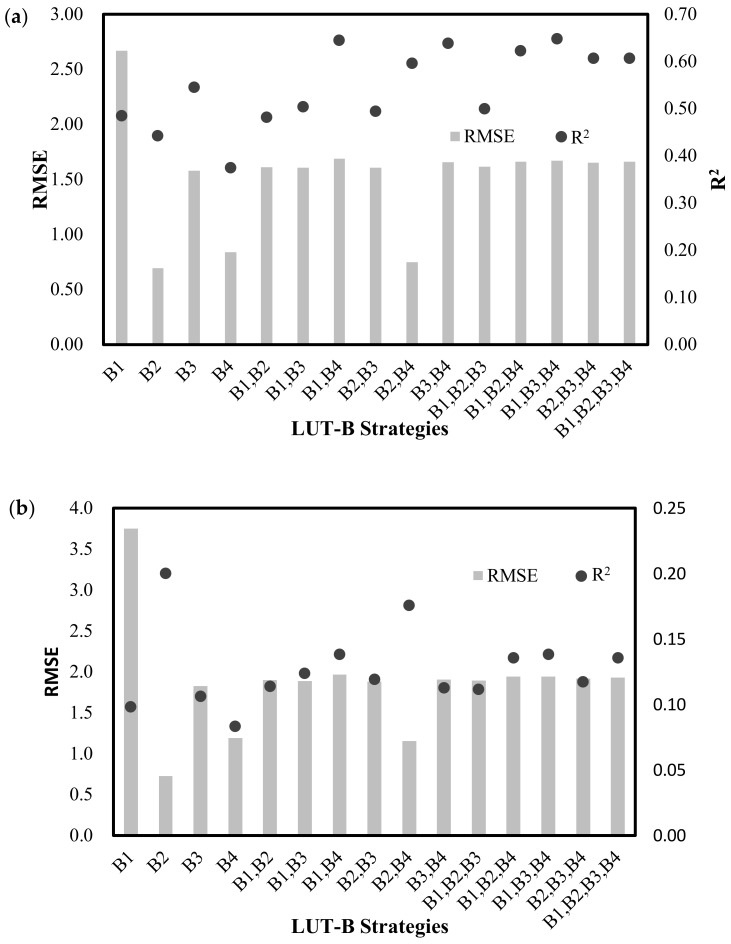
Performances of LAI-LUT strategies with different bands of reflectance to estimate LAI based on the GF-1 data on 14 April (**a**) and 25 May (**b**).

The performances of wheat LAI inversion with different LAI-B strategies were compared by using the R^2^ and RMSE between the measured LAI and the simulated LAI in Figure 4. As we knew that the LUT-B strategy with the highest R^2^ and the lowest RMSE would be the best choice in theory, practically, the optimal results may be difficult to achieve in most cases. The LAI-LUT strategies with different bands of reflectance on 14 April (Figure 4a) demonstrated an inconsistency between the R^2^ and RMSE. Under these conditions, the LAI-Green (LAI-B2) strategy performed best with the lowest RMSE value of 0.69 but did not have the highest R^2^ value (R^2^ = 0.44). Aside from this strategy, the LAI-(Green, Nir) (LAI-(B2, B4)) and LAI-Nir (LAI-B4) strategies also seemed to more correctly estimate the LAI from the GF-1 satellite data, with the RMSE of 0.75 and 0.84, and the R^2^ of 0.60 and 0.38, respectively. Although the rest of LAI-LUT strategies owned a relatively higher R^2^ value (R^2^ > 0.45), the LAI estimates appeared to be unacceptable with all of the RMSE values higher than 1.50. In particular, for strategies with the blue band, LAI-Blue (LAI-B1) performed the worst, with a RMSE and R^2^ of 2.67 and 0.49, respectively. For the LAI-LUT strategies with price function established by band reflectance (R) from the GF-1 data on 25 May (Figure 4b), the LAI-Green strategy also performed the best with the lowest RMSE value of 0.74, and the highest R^2^ of 0.20. 

Next, the LAI-(Green, Nir) showed consistency with a lower RMSE of 1.16 and a higher R^2^ of 0.18. The LAI-Nir strategies showed inconsistency with a lower RMSE of 1.19 and lower R^2^ of 0.08. The rest of the LAI-LUT strategies appeared to be unacceptable with all of the RMSE higher than 1.50, especially for the strategies with the blue band, LAI-Blue (LAI-B1) performed the worst, with a RMSE of 3.7.

These results demonstrated that selecting the proper LAI-reflectance strategy from a LUT approach for the PROSAIL model was needed and played an important role in improving the final accuracy of the LAI estimation. In general, it is essential to establish a LAI-LUT strategy with green and near-infrared bands rather than the blue band to successfully estimate the LAI.

### 4.2. Effects of Different LAI-VI Strategies on LAI Retrieval

**Figure 5:**
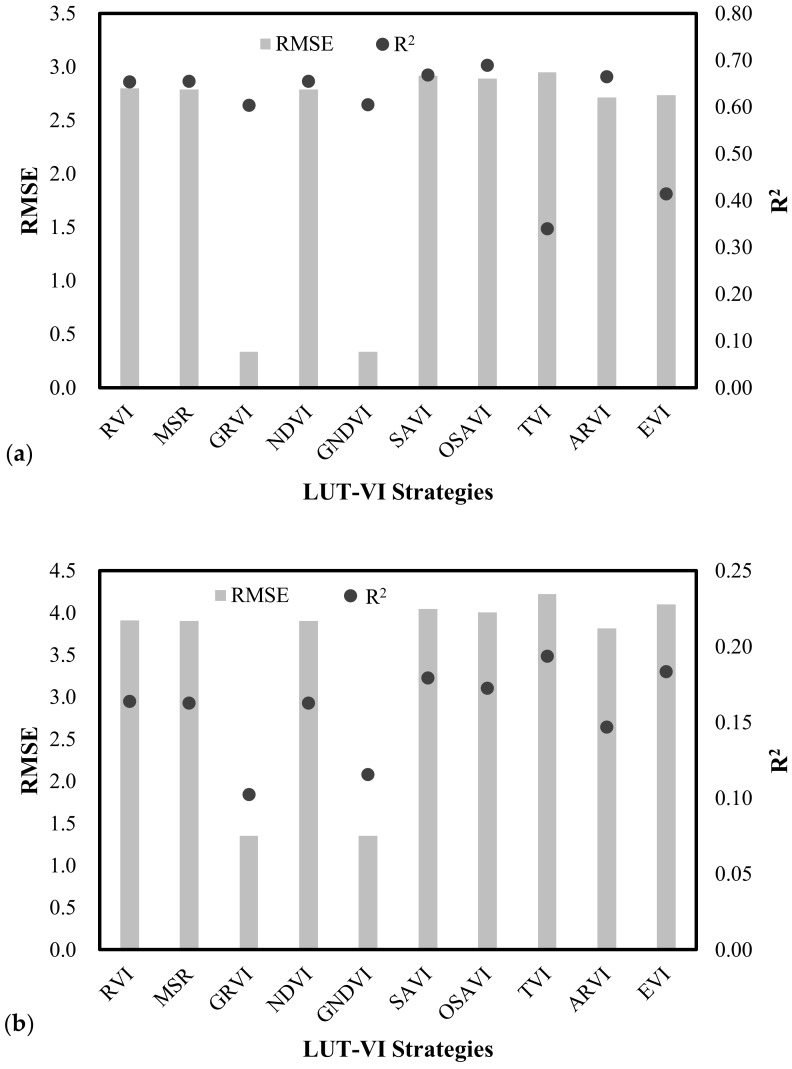
Performances of LAI-LUT strategies with different VIs to estimate the LAI based on the GF-1 data on 14 April (**a**) and at 25 May (**b**).

The performances of wheat LAI inversion with different LAI-VI strategies are compared in Figure 5. The LAI-LUT strategies with different VIs on 14 April (Figure 5a) demonstrated a consistency between the R^2^ and RMSE. Both of the LAI-GNDVI and LAI-GRVI strategies performed best to estimate LAI correctly from the GF-1 satellite data. With the exception of the above two LAI-VI strategies, the rest of the other LAI-VI strategies appeared to be unacceptable with the RMSE values all higher than 1.50, although the R^2^ values were relatively higher (R^2^ > 0.34). For the LAI-LUT strategies with price function established by VIs from the GF-1 data on 25 May (Figure 5b), both the LAI-GNDVI and LAI-GRVI strategies were also the best two strategies and showed inconsistency with the lowest RMSE values of 1.35 and 1.36, and the lower R^2^ of 0.12 and 0.10, respectively. The rest of the LAI-VI strategies appeared to be unacceptable with all of the R^2^ lower than 0.05 and RMSE higher than 3.50.

These results demonstrate that an obvious improvement could be observed when a LAI-LUT was established by using the green and near-infrared bands. According to the reasonability and feasibility of the LAI-LUT strategies, a LUT based on LAI-GNDVI or LAI-GRVI seems to be the optimal choice for estimating the regional winter wheat LAI using the multispectral data derived from GF-1 WFV.

### 4.3. Comparison of LAI-B and LAI-VI Combinations on LAI Retrieval with Different Phenological Stages

To analyze the impacts of LAI inversion with different LAI-B and LAI-VIs strategies in different phonological stages, two sets of GF-1 WFV data were conducted during the elongation (14 April) and grain-filling stages (25 May) of the winter wheat in this study. For 14 April (the elongation stage), the inversion results based on the LAI-Vs strategies owned a higher correlation with the measured LAI and higher accuracies than the results from the LAI-B strategies, in particular, strategies with the LAI-GNDVI strategy had the highest accuracy with a RMSE value of 0.34, and the relatively better R^2^ value of 0.61 (Figure 4a and Figure 5a). The reason was that the LAI-Vs strategies contained not only the sensitive bands to LAI, but also highlighted the difference in band reflectance, and avoided the effects of soil background when the winter wheat was thriving during the elongation stages. In addition to the above strategy, the LAI estimates from the LAI-Green strategy also appeared to be acceptable with RMSE values of 0.69, and R^2^ values of 0.44. While the LAI-B strategies had higher accuracies than the LAI-VIs strategies on 25 May (the grain-filling stage), strategies with the LAI-Green strategy owned the highest accuracy with a RMSE value of 0.74, and the better R^2^ value of 0.20 (Figure 4b and Figure 5b). However, the correlation between the inversion results based on the spectral reflectance/VIs strategies and the measured LAI was not good. The reason for this was that the results of the LAI inversion affected by the grain, gradually yellowing leaves, and soil background, could lead to a lower correlation with the concentrated LAI observations. In addition to the above strategy, the LAI estimates from the LAI-GNDVI strategy also appeared to be unacceptable with the RMSE value of 1.40, and R^2^ value of 0.20. Thus, the LAI-Green strategy is an overall better method for both of these phenological stages.

These results demonstrate that the LAI inversion results were affected by different LAI-LUT strategies during the different stages of crop growth. According to the feasibility of the LAI-LUT strategies, a LUT based on the LAI-GNDVI strategy is an optimal method during the elongation stage, and a LUT based on the LAI-Green strategy is the optimal choice for estimating the winter wheat LAI using the GF-1 data during the grain-filling stage. Ultimately, the LAI-Green strategy is the overall optimal method for both of these phenological stages.

### 4.4. Estimates of Winter Wheat LAI

Based on the results of the above comparison of LAI-B and LAI-VI strategies on LAI retrieval, the LAI-GNDVI and LAI-Green were estimated to invert the regional winter wheat LAI using two GF-1 images during the different phenological stages, respectively.

**Figure 6:**
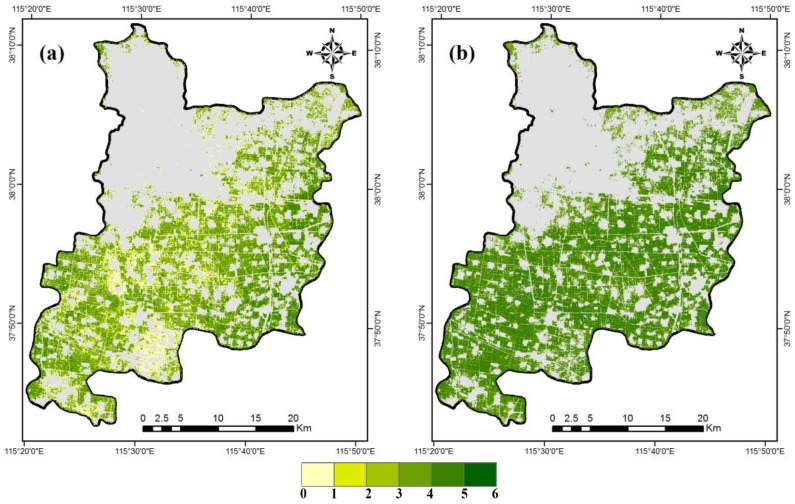
Estimated regional winter wheat LAI maps derived using the LAI-Green and LAI-GNDVI strategies on 14 April (**a**) and 25 May (**b**).

Figure 6 shows the LAI maps from the LAI-GNDVI and LAI-Green strategies derived for the elongation stage and the grain-filling stage. The results of winter wheat LAI inversion during the elongation stage showed that the values of LAI from 0 to 5 could be observed (Figure 6a). The high values appeared in the east of the study region, while the low values appeared in the middle. Due to the late sowing of some winter wheat varieties or drought, some values of wheat LAI had lower LAI values (<2.0) in the same period of elongation stage. The results of LAI inversion during the grain-filling stage demonstrated that LAI values from 0 to 6 could be observed (Figure 6b). For the same period, the LAI values were concentrated between 3.0 and 6.0, and the difference was relatively small between the numerical distributions. The two derived high-resolution LAI maps were in accordance with the actual situations, and good agreement between the estimated and measured LAI value could be observed with the RMSE of 0.34 on 14 April, and RMSE of 0.74 on 25 May.

## 5. Discussion

In this paper, the winter wheat LAI derived from GF-1 WFV images was estimated using the LUT strategies based on the method of PROSAIL RTM. Across all in situ measurements, the effects of different LAI-B strategies and LAI-VIs strategies during the two phenological stages were analyzed and the optimal LUT-LAI strategies for estimating the winter wheat LAI were acquired. Based on the above optimal LUT strategies, ultimate high-resolution LAI maps were obtained, and the values could be observed with the RMSE of 0.34 on 14 April, and 0.74 on 25 May. These results also confirmed that the inversion of PROSAIL RTM using GF-1 WFV data had good potential for estimating the winter wheat LAI with high-resolution. However, some issues still exist and should be addressed to improve its performance.

Several factors would affect the performance of LAI inversion using the GF-1 data. The LUT in generating is one of the key factors, and includes various small factors, e.g., LUT strategies, solution number, LUT sizes, cost function, and the range, distribution and intervals of various variables [73]. To solve the ill-posed problem, an efficient use of prior knowledge improved the accuracy of the variables [37]. Therefore, the parameters of Cab, *C_w_*, *C_m_*, and *ALIA* from the ground measurements were restricted to remove ill-posed results in this study, and the fixations and ranges of other variables in the LUT were got from the previous studies [2,10,25,27,48,68,69,70,71]. Aside from the above parameter settings, the solution number and size of the LUT were also both set as the same as previous studies [9,10,11,32,68]. After that, the generated LUT could achieve an appropriate representation, and trade off the accuracy of the LAI estimates and the computer resources.

In addition, the LUT-LAI strategies also had a great impact on LAI retrieval, and led to the different accuracy of inversion results. In our study, 15 LAI-LUT strategies with the combination of four reflectance bands, and 10 LAI-VIs strategies varied with different results, especially for the LAI-GNDVI or LAI-Green strategies including green and near-infrared bands, or only green band rather than blue band, were the optimal methods for LAI estimation rather than the other strategies with the GF-1 WFV data. The results appeared to be more suitable than the common studies [37] that generated the LUTs with a combination of all reflectance bands. The reason is that the spectral information of some reflectance bands may be unrelated to the LAI, have large noise, or contain lots of repetitive information [9]. The entirely objective function could not be created based on the above reasons. In addition, an extension of two growth stages were chosen to assess the effects of growth variability on winter wheat LAI inversion in our study. According to the feasibility of LAI-LUT strategies, satisfactory results of the LAI estimates were gained by using the LAI-GNDVI strategy during the elongation stage and using the LAI-Green strategy during the grain-filling stage. Ultimately, the LAI-Green strategy is the overall optimal method for both these phenological stages. This finding was similar to the results obtained by Kamal et al. [2], who used Landsat and SPOT5 data for multi-temporal LAI retrievals based on the PROSAIL RTM.

Moreover, LUT-based inversion methods are often strongly affected by measurement uncertainty and simulation noise. When measured LAIs are used to provide and validate remotely sensed LAI estimates, one assumes that the measured LAIs are free of error or assumes that the level of data error is acceptable to be propagated within the simulated PROSAIL RTM [28,49]. To reduce the errors, the measured LAIs were observed using the LAI-2200 Plant Canopy Analyzers, which have proven to be a good alternative in many experimental conditions [14]. However, the observed LAIs are not the “true LAI” but an effective result. Both obey a correlation formula. In this study, the observed LAIs were considered as the “true LAI” since the winter wheat was considered as a turbid medium. Furthermore, the uncertainty effects of these LAI measurements on inverted LAI estimates based on error perturbation will be addressed in our future work. When simulated reflectance/VIs from LUT were used to estimate the remotely sensed LAI, we assumed that the simulated reflectance/VIs were free of error or had low error. To reduce the errors, the measured LAIs, the prior knowledge, solution number and size of the LUT were all considered. In addition, the RRMSE was used as the cost function for uncertainties. However, due to the complexity of the PROSAIL model, simulation uncertainties must exist. Additionally, the uncertainty effects of simulated reflectance/VIs on inverted LAI estimates based on the addition of Gaussian noise [71] will be addressed in our future work.

Nevertheless, good results of LAI were retrieved using the LUT strategies based on the method of PROSAIL RTM from high-resolution GF-1 WFV data with different phenological stages. This study was validated using only two images during the elongation and grain-filling stages; the capabilities regarding the methods presented with other growth stages were not shown. An extension of more growth stages to estimate winter wheat LAI with multi-temporal and multi-resolution sensors will be addressed in our future research.

## 6. Conclusions

Using the LUT-based inversion of the PROSAIL RTM, the performance of LAI estimates from GF-1 WFV data was investigated. The estimation of winter wheat LAI was performed with two phenological stages, elongation and grain-filling, in the study area. Compared to the in situ LAI measurements, the inverted LAIs were evaluated in terms of the R^2^ and RMSE. The effects of different LUT-B and LAI-VIs strategies on LAI retrieval were also explored. The results showed that the LUT strategies of LAI-GNDVI were the optimal choices and had the highest accuracy with the RMSE value of 0.34, and R^2^ value of 0.61 during the elongation stages; and the LUT strategies of LAI-Green were the optimal choices and had the highest accuracy with the RMSE of 0.74, and the R^2^ of 0.20 during the grain-filling stages for the GF-1 WFV data. These results showed that the PROSAIL RTM had great potential for winter wheat LAI retrieval with high accuracy from GF-1 satellite data and selecting the appropriate LUT inversion strategies in different growth periods improved the performance of winter wheat LAI estimation. It is highly significant and valuable research that will contribute to improving the level of agricultural monitoring operation.

## Figures and Tables

**Table 1:** Sensor specifications and calibration coefficients of GF-1 WFV.

Band	Wavelength Range (μm)	Radiometric Resolution (bit)	Spatial Resolution (m)	Swath (km)	Revisit Period (d)	Calibration Coefficients	
						Gain	Offset
Blue (1)	0.45–0.52	10	16	200 (1 CCD) 800 (4 CCD)	4	0.1816	0.00
Green (2)	0.52–0.59					0.1560	0.00
Red (3)	0.63–0.69					0.1412	0.00
Near-infrared (4)	0.77–0.89					0.1368	0.00

**Table 2:** GF-1 images information.

No.	Sensor	Date	*θ_Sensor_*	*ϕ_Sensor_*	*θ_sun_*	*ϕ_sun_*	Time UTC
1	GF-1 WFV1	14 April 2015	63.40	101.44	59.06	154.82	03 h 25 min
2	GF-1 WFV1	25 May 2015	63.31	101.39	70.07	145.89	03 h 26 min

Note: *θ*: zenithal angle; *ϕ*: azimuthal angle.

**Table 3:** A list of vegetation index studied in this study.

No.	Index	Name	Formula	Reference
1	RVI	Ratio VI	RVI = B4/B3	[57]
2	MSR	Modified simple ratio	MSR = (B4/B3 − 1)/(B4/B3 + 1)	[58]
3	GRVI	Green RVI	GRVI = B4/B2 − 1	[59]
4	NDVI	Normalized difference VI	NDVI = (B4 − B3)/(B3 + B4)	[60,61]
5	GNDVI	Green NDVI	GNDVI = (B4 − B2)/(B2 + B4)	[62]
6	SAVI	Soil-adjusted VI	SAVI = (B4 − B3)(1 + L)/(B3 + B4 + L)	[63]
7	OSAVI	Optimization of SAVI	OSAVI = 1.16 * (B4 − B3)/(0.16 + B4 + B3)	[64]
8	TVI	Triangular VI	TVI = 0.5 * (120 * (B4 − B2) − 200 * (B3 − B2))	[65]
9	ARVI	Atmospherically Resistant VI	ARVI = (B4 − B3 − (B1 − B3))/(B4 + B3 − (B1 − B3))	[66]
10	EVI	Enhanced VI	EVI = 2.5 * (B4 − B3)/(B4 + 6.0 * B3 − 7.5 * B1 + 1)	[66,67]

Note: VI represents the vegetation index. B1, B2, B3, B4 represent the bands of Blue, Green, Red, and Near-infrared, respectively.

**Table 4:** Variable combinations for LAI retrieval.

No.	Strategies		No.	Strategies		No.	Strategies
	LAI-B	LAI-VI		LAI-B	LAI-VI		LAI-B
1	B1	RVI	6	B1, B3	SAVI	11	B1, B2, B3
2	B2	MSR	7	B1, B4	OSAVI	12	B1, B2, B4
3	B3	GRVI	8	B2, B3	TVI	13	B1, B3, B4
4	B4	NDVI	9	B2, B4	ARVI	14	B2, B3, B4
5	B1, B2	GNDVI	10	B4, B5	EVI	15	B1, B2, B3, B4

**Table 5:** Ranges and distribution of the leaf, canopy, soil, solar, and sensor parameters in the PROSAIL model.

Parameter	Variables	Unit	Max	Min	Mode	Std.	Type
Leaf	*N_i_*	▬	1.8	1.2	1.5	0.3	Gaussian
	Cab	μg·cm^−2^	75	25	50	7.5	Gaussian
	*C_w_*	cm	0.85	0.60	0.75	▬	Uniform
	*C_m_*	g·cm^−2^	0.011	0.003	0.007	0.002	Gaussian
	*C_bp_*	μg·cm^−2^	0.2	0	0	0.3	Gaussian
Canopy	LAI	▬	8	0	5	▬	Uniform
	*ALIA*	°	80	30	60	4	Gaussian
	*hspot*	▬	0.5	0.1	0.3	0.2	Gaussian
Soil	*psoil*	▬	3.5	0.5	1.2	2.0	Gaussian
Solar & Sensor	*Skyl*	%	▬	▬	10	▬	Fixed
	*tts*	°	70	25	46	▬	Fixed
	*tto*	°	80	0	32	▬	Fixed
	*psi*	°	120	−120	90	▬	Fixed
